# Prediction of Chemotherapy Toxicity After Four Cycles of R‐CHOP Treatment for Diffuse Large B‐Cell Lymphoma: Effective Imaging Biomarkers of Body Composition

**DOI:** 10.1002/cam4.71626

**Published:** 2026-02-25

**Authors:** Yueming An, Liping Zuo, Zhenzhen Jiao, Yuqing Tang, Zhanyu Zhou, Zimeng Yang, Yun Liu, Weiwei Lv, Dexin Yu

**Affiliations:** ^1^ Department of Radiology Qilu Hospital of Shandong University Jinan Shandong China; ^2^ Interdisciplinary Center Shandong University Jinan Shandong China; ^3^ Department of Radiology Shanghe County People's Hospital Jinan Shandong China

**Keywords:** chemotherapy toxicity, computerized tomography, diffuse large B‐cell lymphoma, skeletal muscle

## Abstract

**Background:**

Previous studies have indicated that diffuse large B‐cell lymphoma (DLBCL) patients with a low baseline skeletal muscle (SM) area are more susceptible to severe toxicity during chemotherapy. However, the predictive role of baseline body composition in determining toxicity risk during the initial frontline treatment remains unexplored. This study aims to identify reliable, non‐invasive biomarkers and validate findings using follow‐up CT scans after four cycles of chemotherapy.

**Methods:**

We retrospectively included DLBCL patients who received four cycles of R‐CHOP treatment between January 2015 and January 2024, with pre‐treatment abdominal CT scans. We measured the volume, area, and density of SM, visceral adipose tissue (VAT), and subcutaneous adipose tissue (SAT) to assess their predictive potential for chemotherapy toxicity. Subgroup analyses examined longitudinal changes in body composition, and a logistic regression model identified effective imaging biomarkers associated with grade 3/4 toxicity.

**Results:**

Among the 179 DLBCL patients (mean age 56.96 ± 13.49 years), 46.9% experienced grade 3/4 toxicity. Lower baseline SM volume and density significantly increased the risk of toxicity, particularly in overweight or obese patients (*p* < 0.05). ROC analysis identified SM volume as the best predictor, with a cutoff of 2093.71 cm^3^; patients below this threshold had a 3.34 times higher risk (*p* = 0.001). A decrease in SM volume was associated with higher risks of hematological toxicity (*p* = 0.022) and neutropenic fever (*p* = 0.021).

**Conclusion:**

Lower baseline SM volume and its reductions during treatment are associated with an increased risk of grade 3/4 toxicity, particularly in overweight or obese patients. Body composition measurements serve as effective imaging biomarkers.

## Graphical abstract

Lower baseline skeletal muscle (SM) volume and density are associated with an increased risk of severe toxicity in DLBCL patients. Longitudinal reduction in SM volume during chemotherapy is also an adverse factor.

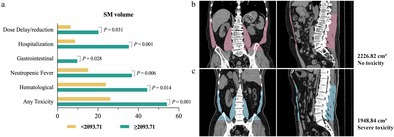

**Abbreviations**
AUC: area under the curve
BMI: body mass index
BSA: body surface area
CI: confidence interval
CT: computed tomography
DLBCL: diffuse large B‐cell lymphoma
HU: hounsfield unit
ICC: intraclass correlation coefficient
L3: the third lumbar vertebra
LBM: lean body mass
LDH: lactate dehydrogenase
OR: odds ratio
R‐CHOP: rituximab, cyclophosphamide, vincristine, doxorubicin and prednisone
ROC: receiver operating characteristic
RR: relative risk
SAT: subcutaneous adipose tissue
SM: skeletal muscle
VAT: visceral adipose tissue

## 1 Introduction

Diffuse large B‐cell lymphoma (DLBCL) is the most common aggressive lymphatic tumor, accounting for approximately 30%–58% of non‐Hodgkin lymphomas [1]. The R‐CHOP regimen comprising rituximab, cyclophosphamide, doxorubicin, vincristine, and prednisone has remained the cornerstone of first‐line therapy for the past two decades, achieving cure rates of 60%–70% [2, 3]. Despite the increased cure rates and extended survival periods, severe chemotherapy‐related toxicity has become a major concern. Chemotherapy can cause both hematologic toxicity, such as bone marrow suppression, and non‐hematologic toxicity, such as febrile neutropenia and gastrointestinal toxicity [4]. These adverse effects not only impact immediate treatment efficacy and reduce patients' quality of life but may also affect long‐term prognosis.

In recent years, skepticism has grown regarding the calculation of chemotherapy drug dosages based on body surface area (BSA). Research indicates that this method inadequately accounts for individual variations in drug clearance, causing some patients to receive incorrect dosages, which can lead to insufficient therapeutic effects or severe toxicity [5, 6]. More precise biomarkers are needed to guide chemotherapy dosages. With CT scans becoming routine for cancer patients, body metrics such as muscle mass and fat distribution can now be accurately measured, making body composition analysis a potential predictive tool [7]. Fat may primarily accommodate lipophilic drugs, while muscle is crucial for the distribution of non‐lipophilic drugs. Variations in body composition can influence the distribution, metabolism, and clearance of anti‐tumor drugs, leading to differences in efficacy and toxicity risks [8].

Previous studies have shown that skeletal muscle mass or density at the third lumbar vertebra (L3) level is associated with long‐term chemotherapy toxicity, suggesting that muscle depletion may elevate toxicity risk in patients with DLBCL [9]. However, such assessments are often delayed and reflect cumulative toxicity rather than early warning. Recent research indicates that reducing R‐CHOP from six to four cycles can maintain therapeutic efficacy while decreasing adverse effects, emphasizing the need to optimize treatment intensity and timing [10, 11] This finding has the potential to influence clinical practices significantly. Therefore, during the initial four cycles, it is critical to adjust dosages based on body composition and implement early interventions to maintain treatment efficacy while minimizing toxicity risks, thereby achieving a balance between effectiveness and safety. Nonetheless, research on predicting early toxicity based on body composition during the initial four cycles is still limited. Further studies are required to optimize DLBCL treatment strategies, reduce toxicity, and improve patient quality of life.

Moreover, weight changes in patients with DLBCL undergoing chemotherapy are frequently observed, typically resulting from both muscle loss and fat deposition [12]. Understanding these changes in body composition during treatment is essential for guiding treatment plans and lifestyle interventions. This study aims to identify effective assessment factors for predicting toxicity after four cycles of chemotherapy and to compare the predictive efficacy of volumetric versus area parameters. By identifying reliable imaging biomarkers of body composition, we aim to bridge the gap in understanding how body composition influences early toxicity during the initial four cycles of chemotherapy.

## 2 Methods

### 2.1 Patients

We established a retrospective cohort of patients diagnosed with DLBCL between January 2015 and January 2024. The inclusion criteria were: (a) a diagnosis of DLBCL confirmed through pathological examination; (b) completion of the initial four‐cycle R‐CHOP treatment and no preventive application of preventive granulocyte colony‐stimulating factor; and (c) availability of clear pre‐chemotherapy abdominal CT scans. Patients were excluded if they had second primary malignancies, special types of lymphoma such as primary central nervous system involvement or transformed lymphoma, or incomplete clinical data. We excluded 16 patients with second primary malignancies, 88 with special types of lymphoma, and 7 with incomplete clinical data. Ultimately, 179 DLBCL patients with CT images (mean age 56.96 ± 13.49 years) were selected. Of these, 74 patients had follow‐up abdominal CT scans after four cycles of chemotherapy, enabling us to observe longitudinal changes in body composition.

### 2.2 Toxicity Assessment

By retrospectively reviewing medical records, we extracted and classified adverse reactions experienced by patients during and after four cycles of chemotherapy, according to the National Cancer Institute Common Terminology Criteria for Adverse Events (NCI‐CTCAE; Version 5.0) [13]. We documented hematological toxicities, including bone marrow suppression indicators such as white blood cell count, absolute neutrophil count, hemoglobin levels, and platelet count, as well as non‐hematological toxicities, such as febrile neutropenia, gastrointestinal toxicity, neurotoxicity, and other chemotherapy‐specific toxicities. Additionally, data were collected on hospitalizations due to chemotherapy toxicity, reductions in chemotherapy drug doses, and delays in treatment.

### 2.3 Clinical Information

Gender, age, and clinical characteristics, including Ann Arbor stage, bone marrow biopsy results, serum lactate dehydrogenase (LDH) levels, Hans classification, and International Prognostic Index, were obtained from each patient's electronic medical records. Upon admission, patients were uniformly dressed in thin hospital gowns, and their height and weight were measured on an empty stomach. Body mass index (BMI) and BSA were calculated using the following formulas [14]:

$$\mathrm{BMI}\;\left(\mathrm{kg}/m^{2}\right)=\text{Weight}\;\left(\mathrm{kg}\right)/{\left(\text{Height}\;\left(m\right)\right)}^{2}$$

$$\mathrm{BSA}\;\left(m^{2}\right)=\sqrt{\;\frac{\text{Height}\;\left(\mathrm{cm}\right)\times \text{Weight}\;\left(\mathrm{kg}\right)}{3600}}$$

We also collected the patients' weight after four cycles of chemotherapy and calculated the longitudinal change in BMI, recorded as ΔBMI.

### 2.4 CT Protocols

Abdominal plain CT scans were conducted using a 64‐slice spiral CT scanner (Siemens Healthcare, Definition AS, Germany). The scan settings included: a tube voltage of 120 kV, tube current set at 150 mA, a pitch of 1 mm, with a layer pitch and thickness each at 5 mm, and a tube rotation duration of 0.5 s. During the scan, patients were positioned supine and instructed to hold their breath.

### 2.5 Body Composition Analysis

**FIGURE 1:**
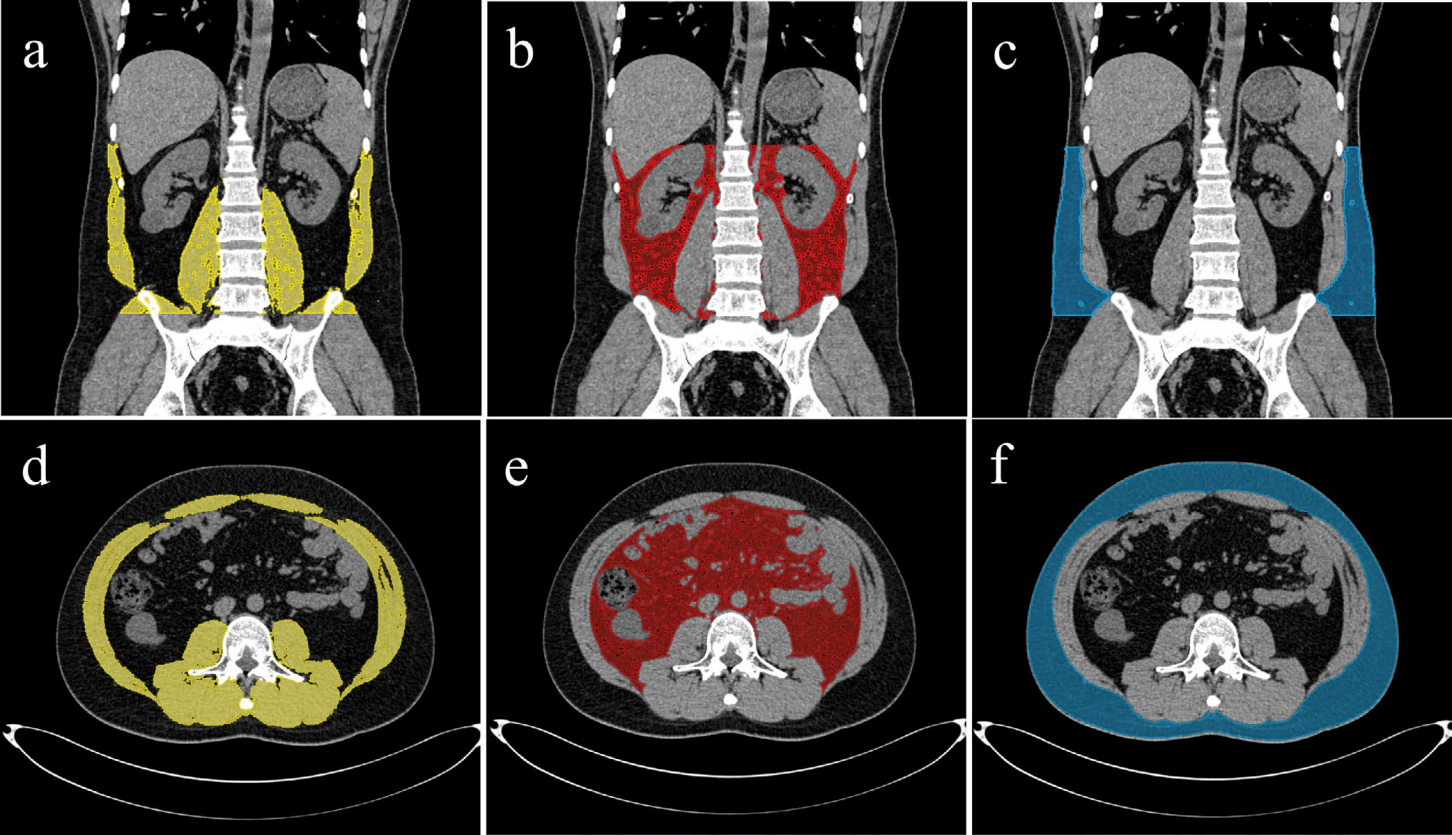
Assessment of abdominal fat and muscle compositions using 3D Slicer software. Images (a, b, and c) display axial CT scans, highlighting the measured volumes and densities. Images (d, e, and f) depict axial CT scans, emphasizing the measured areas and densities, with skeletal muscle marked in yellow, visceral adipose tissue in red, and subcutaneous adipose tissue in blue.

We utilized 3D Slicer software (version 4.10.2, National Institutes of Health, USA) for semi‐automatic recognition of body composition. Thresholds were established based on the standard Hounsfield unit (HU) ranges for skeletal muscle (−29 to +150), subcutaneous fat (−190 to −30), and visceral fat (−150 to −50) [15]. The volumes and average CT densities of SM, VAT, and SAT were measured from levels L1 to L5, while the areas and average CT densities were specifically assessed at level L3, due to its high correlation with body composition volume (Figure 1) [16]. Lean body mass (LBM) was calculated using the following formula [9].

$$\mathrm{LBM}\;\left(\mathrm{kg}\right)=0.3\times \mathrm{SM}\;\text{area}\;\left(cm^{2}\right)+6.06$$

Moreover, we calculated the longitudinal changes in body composition volume and area over the four cycles of chemotherapy, expressed as ΔSM volume, ΔSM area, ΔVAT volume, ΔVAT area, ΔSAT volume, and ΔSAT area.

### 2.6 Statistical Analysis

Continuous variables are presented as mean ± standard deviation, while categorical variables are expressed as frequency (percentage). For continuous variables with a normal distribution, an independent sample *t*‐test was employed, whereas continuous variables with a non‐normal distribution were analyzed using the Mann–Whitney U test. The chi‐square test was utilized for categorical variables. Inter‐observer reproducibility of the volume, area, and average CT densities of SM, VAT, and SAT was assessed using the intraclass correlation coefficient (ICC) and 95% CI. The ICC values were interpreted as follows: poor (0.00 to 0.49), moderate to good (0.50 to 0.74), and excellent (0.75 to 1.00). In our study, all ICC values were ≥ 0.92, indicating excellent consistency.

Initially, we employed Pearson tests to determine whether there was a linear correlation between the volume and area indices of body composition (SM, VAT, SAT) and BMI, BSA, and LBM. Subsequently, we developed two models to report the ORs and 95% CIs for associations between body composition measurements and toxicity outcomes: Model 1 was unadjusted for clinical factors, while Model 2 was adjusted for age, BMI, BSA, LDH, Ann Arbor stage, Hans classification, and International Prognostic Index at diagnosis. We also generated ROC curves and calculated the area under the curve (AUC) to assess the predictive capabilities of each body composition measurement. The Youden Index was used to determine the optimal cutoff point for SM volume to maximize sensitivity and specificity. Relative risks (RRs) were calculated between groups above and below this cutoff point. Additionally, we explored the relationship between SM volume parameters and chemotherapy toxicity across different BMI populations through subgroup analyses and estimated the nonlinear risk between them using a four‐knotted restricted cubic spline regression. Finally, the ORs and 95% CIs associated with longitudinal changes in body composition over four cycles and their relationship to toxicity outcomes were reported. Participants were divided into four groups based on BMI, according to the World Health Organization standard classification for weight status: underweight (BMI < 18.5 kg/m^2^), normal (BMI 18.5 ~ 24.9 kg/m^2^), overweight (BMI 25.0 ~ 29.9 kg/m^2^), and obese (BMI > 30.0 kg/m^2^). Due to the insufficient number of individuals categorized as underweight or obese, we combined underweight and normal‐weight participants into a BMI < 25.0 group and overweight and obese participants into a BMI ≥ 25.0 group.

The sample size for this study was determined based on the initial study design and was set to be five, ten, or twenty times the number of variables in the logistic regression analysis model to minimize the risk of overfitting. Data management and analysis were conducted using SPSS statistical software (Version 25.0; IBM Corp., Armonk, NY, USA) and GraphPad Prism 9.5.1 (La Jolla, CA, USA). Restricted cubic splines were plotted using R software (version 4.2.2, R Foundation for Statistical Computing). All analyses were conducted with a two‐tailed significance level set at less than 0.05 as the threshold for statistical significance.

This study was approved by the Research Ethics Committee, and written informed consent was not required (KYLL‐202308‐038).

## 3 Results

### 3.1 Imaging Baseline Body Composition Characteristics and Clinical Information of the Study Population

Between January 2015 and January 2024, out of 1071 patients diagnosed with DLBCL at our hospital, 328 underwent the initial four‐cycle R‐CHOP chemotherapy regimen, with 290 having clear pre‐chemotherapy abdominal CT scans. Ultimately, 179 DLBCL patients were included, 48.6% of whom were male, with an average age of 56.96 ± 13.49 years (Figure S1). About 46.9% experienced grade 3/4 chemotherapy toxicity, primarily hematological, followed by febrile neutropenia. Other grade 3/4 reactions included rash, fatigue, chills, hepatic and renal impairment, and oral ulcers. No significant differences in age, BMI, BSA, Ann Arbor stage, LDH levels, Hans classification, International Prognostic Index, diabetes mellitus, or hyperlipidemia were observed between groups with and without grade 3/4 toxicity (*p* > 0.05). However, baseline SM volume and density were significantly lower in the toxicity group (1643.84 vs. 1812.06, *p* = 0.025; 37.46 vs. 39.68, *p* = 0.038). Detailed baseline characteristics are available in (Table S1).

### 3.2 Correlation Between Imaging Baseline Body Composition Indices and BMI and BSA

**FIGURE 2:**
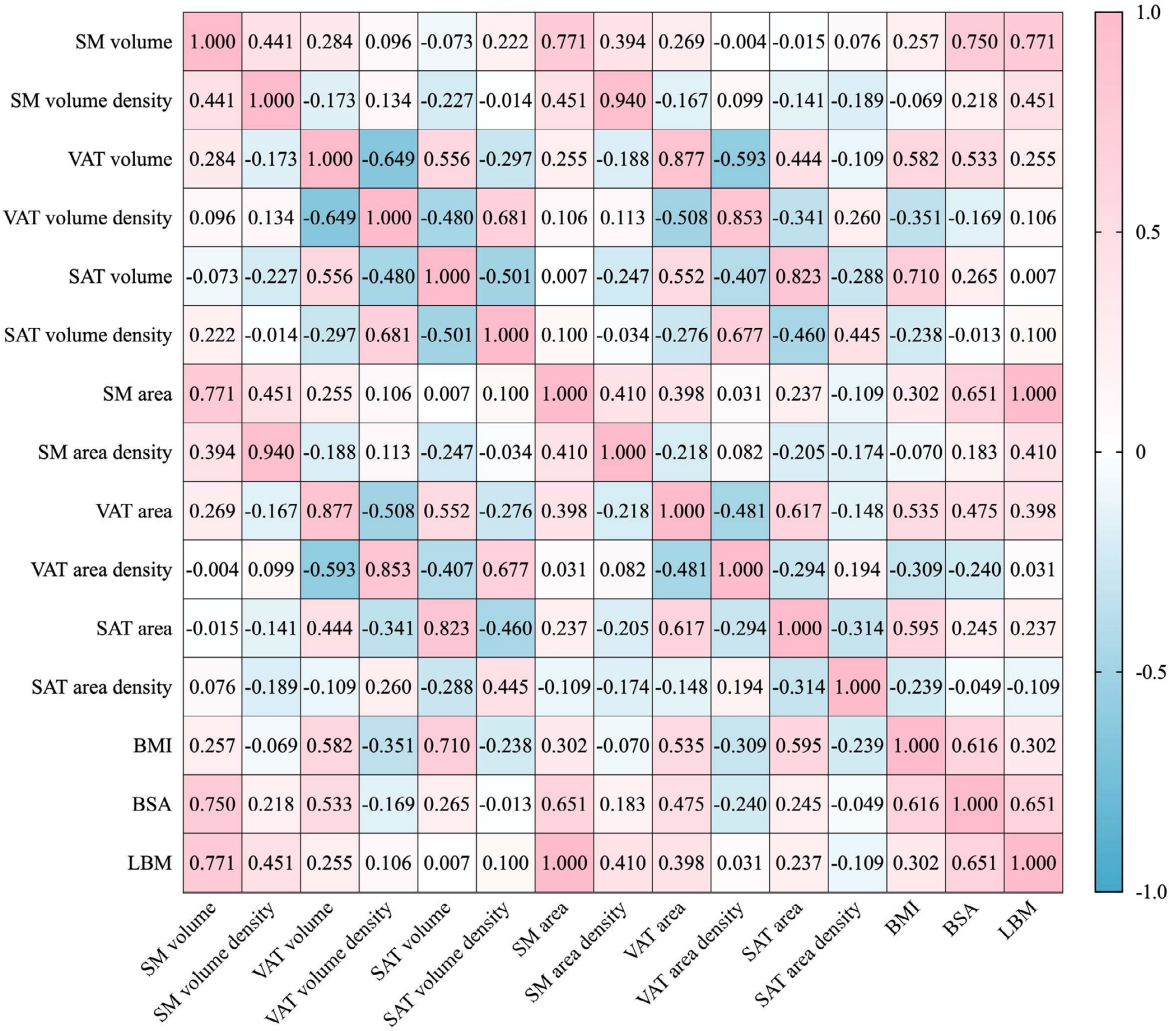
Correlations between body composition parameters in DLBCL patients. The figure shows the correlation matrix between various body composition parameters, including skeletal muscle (SM), visceral adipose tissue (VAT), subcutaneous adipose tissue (SAT), body mass index (BMI), body surface area (BSA), and lean body mass (LBM). Notably, there is a strong positive correlation between SM volume and SM area (*r* = 0.771), and an extremely high correlation between SM volume density and SM area density (*r* = 0.940), highlighting the strong relationship between these volume and area measurements.

Correlational analysis was employed to explore the relationships between baseline body composition volumes, area parameters, BMI, BSA, and LBM in DLBCL patients. A strong correlation was observed between baseline SM volume and SM area (r_s_ = 0.77, *p* < 0.001), as well as between the densities of baseline SM volume and SM area (r_s_ = 0.94, *p* < 0.001). The correlations between BMI and both baseline SM volume and SM area were relatively weak (r_s_ = 0.26, *p* < 0.001; r_s_ = 0.30, *p* < 0.001). Other specific correlation results are illustrated in (Figure 2).

### 3.3 Imaging Baseline Body Composition Predicts Grade 3/4 Chemotherapy Toxicity After Four Cycles in Patients With DLBCL

(Table 1) presents the odds ratio (OR) and 95% confidence interval (CI) for the association between baseline body composition parameters and the risk of grade 3/4 toxicity in an unadjusted logistic regression model. For every 100‐unit decrease in baseline SM volume, the risk of any toxicity increases by 7% (OR 1.07, 95% CI 1.01, 1.14, *p* = 0.028). Similarly, for every 5‐unit decrease in baseline SM volume density, the risk increases by 25% (OR 1.25, 95% CI 1.01, 1.54, *p* = 0.040). Other factors such as baseline SM area, BMI, BSA, lean body mass (LBM), and SAT/VAT volume and density were not associated with grade 3/4 toxicity. After adjusting for age, BMI, BSA, LDH, Ann Arbor stage, Hans classification, and International Prognostic Index, the association between baseline SM density and toxicity risk remained significant (OR 1.30, 95% CI 1.00, 1.69, *p* = 0.047) (Table 2).

**TABLE 1:** Unadjusted ORs (95% CIs) of toxicity for body composition measures.

	Any grade 3/4 toxicity (*N* = 84)	Grade 3/4 hematologic toxicity (*N* = 70)	Grade 3–4 neutropenia fever (*N* = 56)	Grade 3/4 gastrointestinal toxicity (*N* = 13)	Hospitalization (*N* = 51)	Dose delay/reduction (*N* = 20)
BMI (1 kg/m^2^ decrease)	1.05 (0.95, 1.15)	1.02 (0.92, 1.12)	1.03 (0.93, 1.14)	1.01 (0.84, 1.21)	1.08 (0.97, 1.21)	1.06 (0.93, 1.20)
BSA (1m^2^ decrease)	3.36 (0.52, 21.82)	2.60 (0.39, 17.49)	7.69 (0.98, 60.71)	1.85 (0.05, 63.90)	17.96 (2.01, 160.64)*	9.66 (0.74, 126.74)
LBM (5 kg decrease)	1.08 (0.99, 1.19)	1.06 (0.97, 1.15)	1.12 (0.98, 1.28)	1.13 (0.83, 1.53)	1.13 (0.97, 1.30)	1.07 (0.93, 1.22)
**SM**						
SM volume (100 cm^3^ decrease)	1.07 (1.01, 1.14)*	1.04 (0.98, 1.10)	1.09 (1.01, 1.16)*	1.11 (0.97, 1.27)	1.15 (1.06, 1.24)***	1.17 (1.06, 1.29)**
SM volume density (5 HU decrease)	1.25 (1.01, 1.54)*	1.24 (1.00, 1.54)*	1.28 (1.02, 1.61)*	1.09 (0.73, 1.61)	1.18 (0.93, 1.48)	1.39 (1.05, 1.85)*
SM area (1 unit decrease)	1.03 (1.00, 1.05)	1.02 (0.99, 1.04)	1.03 (0.99, 1.08)	1.04 (0.95, 1.14)	1.04 (0.99, 1.08)	1.02 (0.98, 1.06)
SM area density (1 unit decrease)	1.02 (0.98, 1.06)	1.03 (0.99, 1.07)	1.04 (1.00, 1.09)	1.00 (0.93, 1.08)	1.03 (0.98, 1.07)	1.05 (1.00, 1.11)
**VAT (1 unit decrease)**						
VAT volume	1.00 (1.00, 1.00)	1.00 (1.00, 1.00)	1.00 (1.00, 1.00)	1.00 (1.00, 1.00)	1.00 (1.00, 1.00)	1.00 (1.00, 1.00)
VAT volume density	1.01 (0.99, 1.03)	1.01 (0.99, 1.03)	1.01 (0.98, 1.04)	1.01 (0.98, 1.03)	1.00 (0.98, 1.02)	1.02 (0.98, 1.07)
VAT area	1.02 (1.00, 1.04)	1.01 (0.99, 1.03)	1.02 (0.99, 1.05)	1.08 (0.98, 1.19)	1.03 (1.00, 1.07)	1 (0.971.02)
VAT area density	1.01 (0.99, 1.03)	1.01 (0.99, 1.03)	1.01 (0.99, 1.03)	1.01 (0.99, 1.03)	1.00 (0.98, 1.02)	1.03 (0.99, 1.08)
**SAT (1 unit decrease)**						
SAT volume	1.00 (1.00, 1.00)	1.00 (1.00, 1.00)	1.00 (1.00, 1.00)	1.00 (1.00, 1.00)	1.00 (1.00, 1.00)	1.00 (1.00, 1.00)
SAT volume density	1.01 (0.98, 1.04)	1.01 (0.98, 1.04)	1.02 (0.98, 1.05)	1.05 (1.00, 1.10)	1.01 (0.98, 1.05)	1.01 (0.98, 1.05)
SAT area	1.02 (0.99, 1.04)	1.01 (0.99, 1.03)	1.01 (0.99, 1.03)	1.03 (0.97, 1.10)	1.01 (0.99, 1.03)	1.01 (0.98, 1.04)
SAT area density	1.00 (0.98, 1.03)	1.01 (0.98, 1.03)	1.01 (0.98, 1.04)	1.01 (0.96, 1.06)	1.03 (1.00, 1.07)	1.01 (0.98, 1.04)

Abbreviations: BMI, body mass index; BSA, body surface area; CI, confidence interval; LBM, lean body mass; OR, odds ratio; SAT, subcutaneous adipose tissue; SM, skeletal muscle; VAT, visceral adipose tissue.

*** *p* < 0.001.

** *p* < 0.01.

* *p* < 0.05.

**TABLE 2:** Adjusted ORs (95% CIs) of toxicity for body composition measures.

	Any grade 3/4 toxicity (*N* = 84)	Grade 3/4 hematologic toxicity (*N* = 70)	Grade 3–4 neutropenia fever (*N* = 56)	Grade 3/4 gastrointestinal toxicity (*N* = 13)	Hospitalization (*N* = 51)	Dose delay/reduction (*N* = 20)
SM volume (100 cm^3^ decrease)	1.11 (1.00, 1.24)	1.02 (0.91, 1.13)	1.06 (0.94, 1.18)	1.45 (1.13, 1.85)**	1.19 (1.04, 1.37)*	1.17 (1.06, 1.29)**
SM volume density (5 HU decrease)	1.30 (1.00, 1.69)*	1.30 (1.00, 1.69)*	1.26 (0.96, 1.66)	1.13 (0.70, 1.80)	1.12 (0.85, 1.49)	1.38 (0.98, 1.95)

*Note:* Model 1: Adjusted for age, BMI, BSA, LDH, Ann Arbor stage, GCB·or non‐GCB, and·International Prognostic Index.

Abbreviations: CI, confidence interval; OR, odds ratio; SM, skeletal muscle.

** *p* < 0.01.

* *p* < 0.05.

**FIGURE 3:**
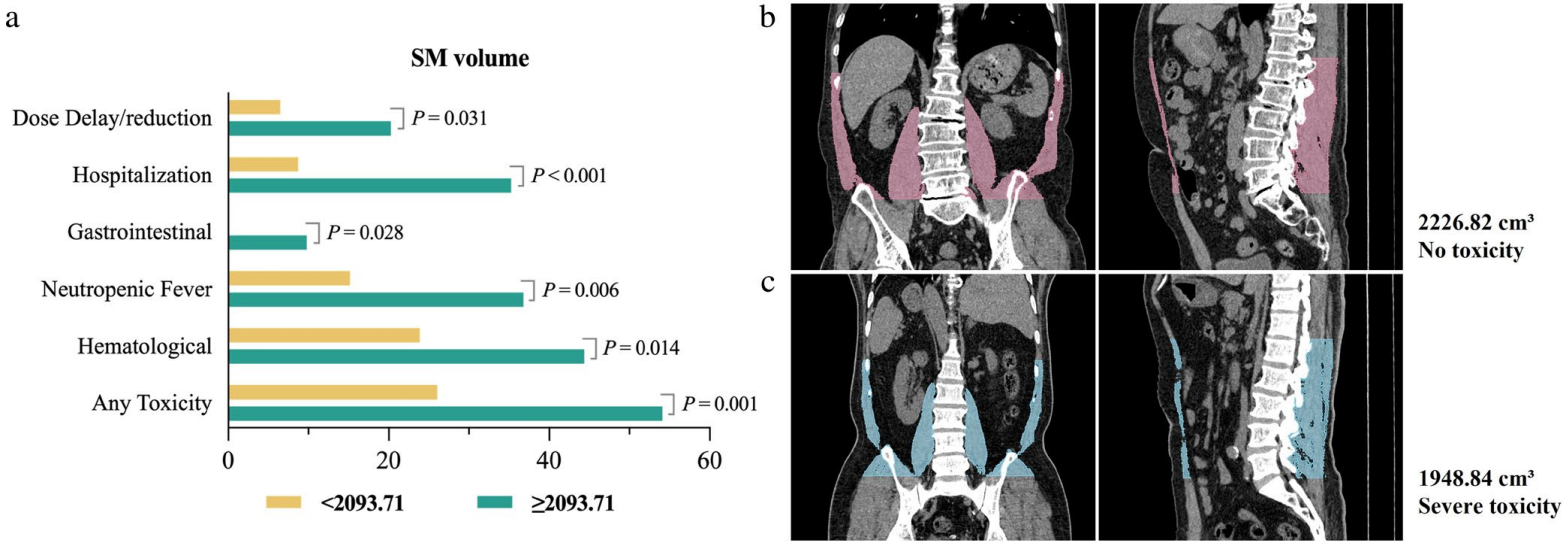
(a) illustrates the risk of grade 3/4 toxicity calculated based on SM volume, with the P value obtained from logistic regression. (b) shows a 73 year‐old male with a baseline SM volume of 2226.82 cm^3^, above the threshold, who did not experience toxicity. In contrast, (c) presents a 74 year‐old male with a baseline SM volume of 1948.84 cm^3^, below the threshold, who experienced grade 3/4 toxic events.

We utilized ROC curves and AUC to analyze the relationship between baseline BMI, BSA, SM volume, and area parameters with any grade 3/4 toxicity (Figure S2). All parameters showed good predictive ability, with baseline SM volume being the best predictor (AUC = 0.60). The optimal cutoff for baseline SM volume was determined to be 2093.71 cm^3^ using the Youden index (Figure 3a), illustrates the probability differences in various grade 3/4 toxicity events when patients were divided based on this cutoff. Patients with lower baseline SM volume were more likely to experience toxicity (*p* = 0.001).

Further analysis indicated that patients with lower baseline SM volumes had a threefold higher risk of experiencing any toxicity compared to those with higher volumes (RR 3.34, 95% CI 1.59, 7.02, *p* = 0.001). For instance (Figure 3b), shows a 73 year‐old male with a baseline SM volume of 2226.82 cm^3^ (> 2093.71 cm^3^) who did not experience any chemotherapy‐related toxicities. In contrast (Figure 3c), depicts a 74 year‐old male with a baseline SM volume of 1948.84 cm^3^ (< 2093.71 cm^3^) who experienced grade 3/4 toxic events. This underscores the significance of the baseline SM volume threshold in predicting chemotherapy toxicity risk.

### 3.4 Imaging Baseline Body Composition Predicts Grade 3/4 Hematologic Toxicity After Four Cycles in Patients With DLBCL

A total of 70 patients (39.1%) experienced grade 3/4 hematologic toxicity during the four‐cycle R‐CHOP chemotherapy regimen. A lower baseline SM volume density was significantly associated with a higher risk of hematologic toxicity (OR 1.24, 95% CI 1.00, 1.54, *p* = 0.049). However, after adjusting for all baseline clinical factors, although not statistically significant, every 5‐unit decrease in baseline SM volume density was associated with an increased risk of hematologic toxicity (OR 1.30, 95% CI 1.00, 1.69, *p* = 0.052). Bone marrow biopsy results indicated that 13 out of 179 patients had bone marrow involvement by lymphoma prior to treatment. Of these, 6 patients experienced grade 3/4 hematological toxicities, while 7 patients did not exhibit such toxicities. Chi‐square testing showed no significant association between bone marrow infiltration and the occurrence of grade 3/4 hematologic toxicity (*p* = 0.589).

### 3.5 Imaging Baseline Body Composition Predicts Grade 3/4 Neutropenia Fever and Gastrointestinal Toxicity After Four Cycles in Patients With DLBCL

During the four cycles of R‐CHOP chemotherapy, 56 patients (30.7%) experienced grade 3/4 febrile neutropenia, while 13 (10.6%) had severe gastrointestinal reactions like nausea and vomiting. Unadjusted analyses indicated that reductions in baseline SM volume and density were significantly associated with febrile neutropenia (OR 1.09, 95% CI 1.01–1.16, *p* = 0.018; OR 1.28, 95% CI 1.02–1.61, *p* = 0.033). However, these associations lost significance after adjusting for all baseline clinical factors. In unadjusted analyses, baseline SM volume and density were not linked to gastrointestinal toxicity. Nevertheless, after adjustments, each 100 cm^3^ reduction in baseline SM volume increased the risk of gastrointestinal reactions by 45% (OR 1.45, 95% CI 1.13–1.85, *p* = 0.003). Further stratification revealed that patients with low SM volume (< 2093.71 cm^3^) had approximately three times the risk of febrile neutropenia compared to those with high SM volume (≥ 2093.71 cm^3^) (RR 3.25, 95% CI 1.35–7.82, *p* = 0.009). There was no significant difference in the incidence of gastrointestinal toxicity between the two groups (*p* > 0.05).

### 3.6 Imaging Baseline Body Composition Predicts Hospitalization and Dose Delays/Reductions in Patients With DLBCL

A total of 51 patients (28.5%) were hospitalized due to toxic events, and 20 patients (14.0%) experienced delays or dose reductions in chemotherapy drugs. In unadjusted analyses, reductions in baseline SM volume were significantly associated with increased risk of hospitalization and dose delays/reductions (OR 1.15, 95% CI 1.06–1.24, *p* < 0.001; OR 1.17, 95% CI 1.06–1.29, *p* = 0.009). These associations remained significant in adjusted analyses. Additionally, in unadjusted analyses, for every 5 HU decrease in baseline SM volume density, the risk of dose delays/reductions increased by 39% (OR 1.39, 95% CI 1.05–1.85, *p* = 0.030), though this association was no longer apparent in the adjusted analyses. Further stratification showed that patients with low baseline SM volume (< 2093.71 cm^3^) had a significantly higher likelihood of being hospitalized due to chemotherapy toxicity (RR 5.74, 95% CI 1.94–16.99, *p* = 0.002) and experiencing chemotherapy drug reductions/delays (RR 3.65, 95% CI 1.05–12.67, *p* = 0.041) compared to those with high SM volume (≥ 2093.71 cm^3^).

### 3.7 Association Between Imaging Baseline SM Volume and Density and Four‐Cycle Chemotherapy Toxicity Among Different BMI Subgroups of Patients With DLBCL

**FIGURE 4:**
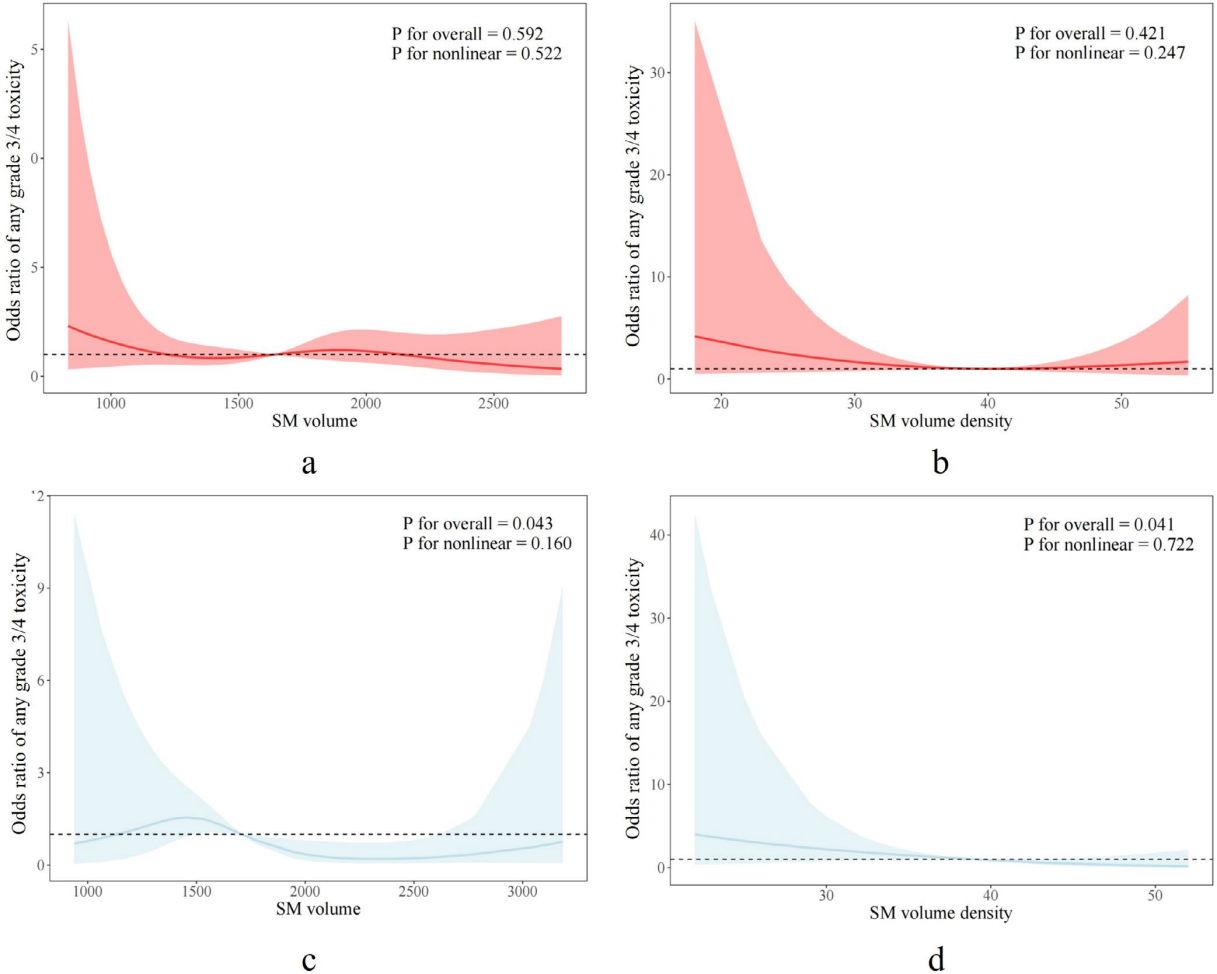
Histograms and line graphs show nonlinear associations between abdominal CT‐based (a) SM volume and (b) SM volume density among DLBCL patients in the low‐BMI group (BMI < 25.0 kg/m^2^) and (c) SM volume and (d) SM volume density in the high‐BMI group (BMI ≥ 25.0 kg/m^2^).

A total of 116 patients with DLBCL were included in the BMI < 25.0 group, and 63 were included in the BMI ≥ 25.0 group. The average baseline SM volume was significantly larger in the BMI ≥ 25.0 group of DLBCL patients compared to the BMI < 25.0 group (1872.49 vs. 1657.42, *p* = 0.017), while the percentage of SM volume out of total fat and muscle, SM%, was significantly lower in the BMI ≥ 25.0 group (26.50 vs. 36.22, *p* < 0.001). The baseline SM density was lower in the BMI ≥ 25.0 group compared to the BMI < 25.0 group, but the difference was not statistically significant (38.91 vs. 38.16, *p* = 0.254). Details of baseline clinical characteristics differences among other BMI subgroups can be found in (Table S2). In the BMI < 25.0 group, there was no significant association between baseline SM volume and density and the risk of grade 3/4 toxicity in DLBCL patients (Figure 4a,b). However, in the BMI ≥ 25.0 group, a lower SM volume and density were linearly negatively correlated with the risk of grade 3/4 toxicity in DLBCL patients (Figure 4c,d).

In the BMI ≥ 25.0 group, without adjustment for baseline clinical factors, low baseline SM volume was significantly associated with an increased risk of any grade 3/4 toxicity, hematological toxicity, neutropenic fever, hospitalization, and dose reduction/delay (*p* < 0.05) (Table 3). In the adjusted analysis, each 100 cm^3^ decrease in baseline SM volume increased the likelihood of any Grade 3/4 toxicity by 30% (OR 1.30, 95% CI 1.07, 1.59, *p* = 0.009), the likelihood of hematological toxicity increased by 23% (OR 1.23, 95% CI 1.02, 1.47, *p* = 0.030), and the risk of dose reduction/delay increased by 37% (OR 1.37, 95% CI 1.02, 1.85, *p* = 0.038). In the BMI < 25.0 group, low baseline SM volume was significantly related to increased risk of hospitalization and dose reduction/delay only in the unadjusted analysis (*p* < 0.05), and the OR values were lower than those in the BMI ≥ 25.0 group (1.11 vs. 1.22; 1.15 vs. 1.23). This association was no longer significant after adjusting for clinical factors.

**TABLE 3:** Unadjusted and adjusted ORs (95% CIs) of toxicity for SM volume measures in the subgroups divided by BMI.

		SM volume (100 cm^3^ decrease)		SM volume density (5 HU decrease)	
		OR	OR1	OR	OR1
BMI < 25.0	Any grade 3/4 toxicity (*N* = 56)	1.04 (0.95, 1.12)	1.02 (0.88, 1.18)	1.09 (0.84, 1.41)	1.12 (0.81, 1.56)
	Grade 3/4 hematologic toxicity (*N* = 45)	1.02 (0.94, 1.10)	1.13 (0.96, 1.32)	1.08 (0.83, 1.41)	1.14 (0.82, 1.59)
	Grade 3–4 neutropenia fever (*N* = 36)	1.04 (0.95, 1.14)	1.02 (0.87, 1.20)	1.12 (0.85, 1.48)	1.14 (0.81, 1.61)
	Grade 3/4 gastrointestinal toxicity (*N* = 8)	1.11 (0.93, 1.32)	1.43 (1.05, 1.94)	1.14 (0.68, 1.90)	1.12 (0.61, 2.05)
	Hospitalization (*N* = 34)	1.11 (1.01, 1.23)*	1.16 (0.98, 1.37)	1.00 (0.76, 1.32)	1.06 (0.75, 1.50)
	Dose delay/reduction (*N* = 18)	1.15 (1.01, 1.30)*	1.18 (0.95, 1.46)	1.22 (0.85, 1.73)	1.15 (0.75, 1.75)
BMI ≥ 25.0	Any grade 3/4 toxicity (*N* = 28)	1.12 (1.01, 1.23)*	1.30 (1.07, 1.59)**	1.67 (1.12, 2.49)*	1.70 (1.06, 2.72)*
	Grade 3/4 hematologic toxicity (*N* = 25)	1.12 (1.01, 1.24)*	1.23 (1.02, 1.47)*	1.65 (1.11, 2.46)*	1.69 (1.04, 2.73)*
	Grade 3–4 neutropenia fever (*N* = 20)	1.16 (1.03, 1.31)*	1.21 (0.99, 1.48)	1.68 (1.11, 2.56)*	1.62 (0.94, 2.73)
	Grade 3/4 gastrointestinal toxicity (*N* = 5)	1.13 (0.92, 1.38)	1.80 (0.97, 3.34)	1.53 (0.79, 2.99)	2.93 (0.72, 11.94)
	Hospitalization (*N* = 17)	1.22 (1.06, 1.40)**	1.25 (0.96, 1.63)	1.67 (1.08, 2.58)*	1.91 (0.99, 3.68)
	Dose delay/reduction (*N* = 12)	1.23 (1.04, 1.46)*	1.37 (1.02, 1.85)*	1.75 (1.07, 2.87)*	2.02 (0.99, 4.12)

*Note:* Model 1: Adjusted for age, BMI, BSA, LDH, Ann Arbor stage, GCB·or non‐GCB, and·International Prognostic Index.

Abbreviations: BMI, body mass index; CI, confidence interval; OR, odds ratio; SM, skeletal muscle.

** *p* < 0.01.

* *p* < 0.05.

Regarding the relationship between baseline SM volume density and grade 3/4 toxicity, in the BMI ≥ 25.0 group, low SM volume density was significantly associated with an increased risk of any grade 3/4 toxicity, hematological toxicity, neutropenic fever, hospitalization, and the risk of dose reduction/delay in the unadjusted analysis (*p* < 0.05). In the adjusted analysis, for every 5 HU decrease in baseline SM volume density, the probability of any grade 3/4 toxicity occurrence increased by 70% (OR 1.70, 95% CI 1.06, 2.72, *p* = 0.026), and the probability of hematological toxicity increased by 69% (OR 1.69, 95% CI 1.04, 2.73, *p* = 0.034). However, in the BMI < 25.0 group, there was no significant correlation between SM volume parameters and the risk of grade 3/4 toxicity.

### 3.8 Changes in Body Composition Over Four Cycles Predict Grade 3/4 Chemotherapy Toxicity in DLBCL Patients

In our study cohort, 74 patients underwent abdominal CT after four cycles of chemotherapy to evaluate treatment effectiveness. Post‐treatment, the average BMI increased by 0.29 kg/m^2^, while the average SM volume decreased by 49.63 cm^3^, with both VAT and SAT volumes increasing—particularly VAT, which rose by 190.45 cm^3^. Areas of SM, VAT, and SAT all decreased to varying extents. Additionally, CT values for SM volume, SAT volume, and SM area showed slight increases, while VAT volume, VAT area, and SAT area CT values decreased, with VAT volume CT value decreasing the most (−7.69 HU) (Table S3). Each 100 cm^3^ decrease in ΔSM volume was associated with a 32% increased risk of grade 3/4 hematologic toxicity (OR 1.32, 95% CI 1.04–1.68, *p* = 0.022) and a 36% increased risk of neutropenia fever (OR 1.36, 95% CI 1.05–1.76, *p* = 0.021). Other longitudinal changes in body composition were not significantly associated with grade 3/4 toxicity (*p* > 0.05) (Table S4).

## 4 Discussion

In this study, we examined the relationship between baseline body composition and severe chemotherapy toxicity in patients with DLBCL during the initial four cycles. Our analysis revealed that baseline SM volume and density can predict toxicity, highlighting their potential as biomarkers. We then followed 74 patients to track changes in body composition after four cycles, finding that decreased SM volume may lead to more severe toxicity. These findings emphasize the importance of assessing body composition before treatment and offer new insights for clinical applications and future research.

Over the past two decades, single‐slice CT has been the reference method for measuring body composition. However, in our toxicity assessment over four chemotherapy cycles, muscle area measurements proved significantly less effective than volumetric measurements for predicting short‐term risks. Studies indicate that differences between slices and errors in estimating total volume from a single slice reduce the accuracy of area measurements [17]. Another longitudinal study confirmed that area measurements poorly predict short‐term body composition changes, exhibiting a weak correlation between area and volume changes [18]. This discrepancy limits the reliable assessment of muscle tissue, potentially leading to inaccurate estimations of drug distribution and increased toxicity risks. Therefore, we emphasize the importance of using SM volume measurements over single‐area measurements for predicting chemotherapy toxicity.

SM may contribute to the heterogeneity of chemotherapy toxicity through its impacts on drug pharmacokinetics, immune function, and treatment tolerance. SM influences drug distribution, metabolism, and clearance. For example, rituximab, a hydrophilic drug, is primarily distributed in skeletal muscle, such that reduced muscle mass may increase plasma concentrations and, consequently, toxicity risks [19]. Prado et al. [20], found that each kilogram reduction in LBM, mainly composed of SM, decreases anthracycline drug clearance by about 19%. This reduction may result from SM loss due to malnutrition and liver degradation, which can diminish the activity and expression of enzymes, especially those mediated by hepatic cytochrome P450. Consequently, reduced liver extraction and drug clearance prolong drug residence time, increasing exposure and the risk of toxic side effects [21]. Additionally, SM supports T‐cell proliferation and immune recovery, so muscle loss may weaken immunity and intensify toxic reactions [22]. SM also produces cytokines like IL‐6, essential for hematopoietic function, so muscle depletion could lead to hematologic toxicity [23]. Furthermore, reduced muscle mass is linked to poor treatment tolerance in DLBCL patients [24, 25]. Therefore, thorough pre‐chemotherapy assessments and individualized dosing are critical to minimizing toxicity risks, further highlighting the importance of SM in chemotherapy outcomes. In this study, we emphasize the relative significance of SM over visceral adipose tissue (VAT) and subcutaneous adipose tissue (SAT) in predicting chemotherapy toxicity. While VAT and SAT play important roles in overall body composition, SM has distinct physiological functions that make it a critical factor in chemotherapy outcomes. Unlike VAT and SAT, which do not offer the same level of metabolic and immune support, SM directly impacts drug pharmacokinetics, immune function, and drug processing, all of which are crucial in reducing chemotherapy toxicity. Therefore, the importance of SM over VAT/SAT lies in its direct involvement in metabolism, immune function, and drug processing—key aspects that help reduce chemotherapy toxicity.

In this study, patients with DLBCL and a BMI ≥ 25.0 exhibited higher SM volume, but their percentage of SM relative to the total mass of fat and muscle (SM%) was significantly lower than that of patients with a BMI < 25.0, suggesting sarcopenic obesity in this group. Sarcopenic obesity is clinically detrimental, as muscles are essential for metabolism, and low SM proportions are linked to poor endurance, slow movement, and higher chemotherapy toxicity risks [26, 27]. This study demonstrates that patients with high SM volumes, but low SM proportions are significantly associated with grade 3/4 toxicity, even after adjusting for baseline factors. These findings underscore the importance of focusing on SM volume and proportion in clinical assessments, rather than solely on BMI or body surface area (BSA), to improve treatment tolerance and health outcomes.

We also observed the effects of chemotherapy drugs on body composition, particularly muscle loss and fat deposition. The reduction in muscle volume during the four cycles of chemotherapy was closely linked to increased chemotherapy toxicity. Common toxicities, such as nausea, vomiting, fatigue, and loss of appetite, negatively affect diet and activity levels, leading to significant muscle loss [28]. Additionally, chemotherapy may activate the degradation of myofibrillar proteins through the ubiquitin‐proteasome pathway or interfere with Insulin‐like Growth Factor 1 activity, affecting muscle synthesis [19]. This reduction in muscle mass threatens patients' physiological health and may adversely affect treatment outcomes. Our findings are consistent with previous research indicating that patients who maintain or increase muscle mass cope better with treatment, while those who experience muscle loss may face rapid disease progression and shorter survival [29]. Therefore, monitoring muscle mass changes during chemotherapy is essential for mitigating toxic side effects and enhancing patient prognosis. Developing interventions to address chemotherapy‐induced muscle loss is a vital area for future research.

While our findings focus on the role of body composition in predicting chemotherapy toxicity, other factors may also contribute to the severity of toxicities observed in patients with DLBCL. For example, bone marrow infiltration by lymphoma can directly impact hematological toxicity by impairing normal bone marrow function, leading to a reduced ability to produce blood cells and thereby exacerbating the effects of chemotherapy. Our analysis showed that although bone marrow involvement by lymphoma was present in 13 patients, we did not observe a significant association between bone marrow infiltration and grade 3/4 hematological toxicities. However, it is essential to consider that the impact of bone marrow infiltration may vary depending on the extent of infiltration and other underlying patient factors. Additionally, concomitant medications such as antivirals or other immunosuppressive agents may affect the severity of chemotherapy toxicity by interacting with chemotherapy drugs and potentially altering their metabolism or clearance. Other supportive medications might also contribute to adverse reactions through similar mechanisms. Although these factors were not specifically controlled for in this study, they remain important considerations for future research to better understand the complexity of chemotherapy toxicity in DLBCL patients.

This study has several limitations. First, its retrospective design may introduce unavoidable bias in patient selection. Second, because the sample is from a single center, it lacks robust external validation. Future prospective studies are needed to confirm these findings in a broader patient population. Third, the limited number of patients with DLBCL prevented the refinement of critical points for SM volume and density by gender or age. Lastly, most participants lacked long‐term follow‐up data on mortality or disease recurrence, which limits a comprehensive assessment of the relationship between body composition measurements and clinical outcomes.

As the survival rate of patients with DLBCL improves, identifying those at high risk of early chemotherapy toxicity becomes crucial. This study demonstrates that lower baseline SM volume and density are significantly linked to a higher risk of severe toxicity. These findings highlight the limitations of traditional BMI and BSA measurements, which may not adequately reflect changes in SM and adipose tissue, thus impacting the accuracy of toxicity predictions. Longitudinal reductions in SM volume are closely associated with higher toxicity risks. Our research supports the use of SM volume and density measurements over area measurements to more accurately predict chemotherapy toxicity, potentially enhancing treatment outcomes and patient quality of life.

## Author Contributions

**Yueming An:** conceptualization (equal), methodology (equal), writing – original draft (equal). **Liping Zuo:** investigation (equal), writing – review and editing (equal). **Zhenzhen Jiao:** data curation (equal). **Yuqing Tang:** formal analysis (equal). **Zhanyu Zhou:** software (equal). **Zimeng Yang:** investigation (equal). **Yun Liu:** resources (equal). **Dexin Yu:** supervision (equal). **Weiwei Lv:** investigation (equal), methodology (equal), writing – original draft (equal), writing – review and editing (equal).

## Funding

The authors have nothing to report.

## Ethics Statement

Approval of the research protocol by an Institutional Reviewer Board. This study was approved by the Research Ethics Committee of Qilu Hospital of Shandong University, and written informed consent was not required (KYLL‐202308‐038).

## Conflicts of Interest

The authors declare no conflicts of interest.

## Supporting information


**Table S1:** Baseline characteristics of patients with DLBCL.
**Table S2:** Baseline characteristics of patients with DLBCL by BMI.
**Table S3:** Body composition characteristics of 74 patients with DLBCL at baseline and after four cycles of chemotherapy.
**Table S4:** ORs (95% CIs) of toxicity for changes in body composition after four cycles of chemotherapy.
**Figure S1:** Selection of participants for the current research.
**Figure S2:** ROCs for different body composition measures and any grade 3/4 toxicities.

## Data Availability

Data sharing not applicable ‐ no new data generated, or the article describes entirely theoretical research.
