# Supplementary material for: Prediction of Chemotherapy Toxicity After Four Cycles of R‐CHOP Treatment for Diffuse Large B‐Cell Lymphoma: Effective Imaging Biomarkers of Body Composition
Source: Cancer Med. 2026 Feb 25;15(3):e71626. doi: 10.1002/cam4.71626 (PMC12935528; doi:10.1002/cam4.71626)
Supplement: Supplementary file 1 — Table S1: Baseline characteristics of patients with DLBCL. Table S2: Baseline characteristics of patients with DLBCL by BMI. Table S3: Body composition characteristics of 74 patients with DLBCL at baseline and after four cycles of chemotherapy. Table S4: ORs (95% CIs) of toxicity for changes in body composition after four cycles of chemotherapy. Figure S1: Selection of participants for the current research. Figure S2: ROCs for different body composition measures and any grade 3/4 toxicities. [file CAM4-15-e71626-s001.docx]

Supplementary Table S1. Baseline characteristics of patients with DLBCL.

| Variables | N (%) / mean (SD) | Group with any grade 3/4 toxicity | Group without any grade 3/4 toxicity | *P* |
| --- | --- | --- | --- | --- |
| No. patients | 179 | 84 | 95 | - |
| **Patient characteristics** |  |  |  |  |
| Age, years | 56.96±13.49 | 57.69±13.51 | 56.31±13.51 | 0.371^b^ |
| BMI, kg/m^2^ | 24.06±3.11 | 23.82±3.39 | 24.26±2.84 | 0.346^a^ |
| BSA, m^2^ | 1.74±0.16 | 1.73±0.17 | 1.76±0.15 | 0.205^a^ |
| Ann Arbor stage |  |  |  | 0.137^c^ |
| I-II | 70(39.1%) | 28(33.3%) | 42(44.2%) |  |
| III-IV | 109(60.9%) | 56(66.7%) | 53(55.8%) |  |
| LDH>UPN | 102(57.0%) | 45(53.6%) | 57(60.0%) | 0.386^c^ |
| GCB/non-GCB | 73/179 | 36/48 | 37/58 | 0.595^c^ |
| International Prognostic Index |  |  |  | 0.444^c^ |
| Low-risk (0-2 points) | 153(85.5%) | 70(83.3%) | 83(87.4%) |  |
| High-risk (3-5 points) | 26(14.5%) | 14(16.7%) | 12(12.6%) |  |
| Diabetes Mellitus | 17(9.5%) | 10(11.9%) | 7(7.4%) | 0.302^c^ |
| Hyperlipidemia | 4(2.2%) | 1(1.2%) | 3(3.2%) | 0.374^c^ |
| **Multi-slice body composition from L1 to L5(means)** |  |  |  |  |
| SM volume, cm^3^ | 1733.11±506.78 | 1643.84±478.63 | 1812.06±520.22 | 0.025^b^ |
| SM volume density, HU | 37.88±11.65 | 37.46±7.41 | 39.68±6.81 | 0.038^a^ |
| VAT volume, cm^3^ | 1853.20±838.66 | 1803.32±844.83 | 1897.30±835.15 | 0.456^a^ |
| VAT volume density, HU | -83.43±16.89 | -84.74±22.42 | -87.22±9.65 | 0.833^b^ |
| SAT volume, cm^3^ | 2097.34±1006.38 | 2052.34±976.11 | 2137.13±1035.93 | 0.575^a^ |
| SAT volume density, HU | -89.09±9.86 | -98.42±10.66 | -97.81±9.14 | 0.485^b^ |
| **Single-slice body composition at mid-L3(means)** |  |  |  |  |
| SM area, cm^2^ | 16.66±14.18 | 14.50±8.73 | 18.57±17.48 | 0.354^b^ |
| SM area density, HU | 39.14±7.36 | 38.54±7.45 | 39.67±7.28 | 0.303^a^ |
| VAT area, cm^2^ | 17.19±18.67 | 14.60±9.68 | 19.49±23.79 | 0.756^b^ |
| VAT area density, HU | -88.07±17.39 | -89.04±10.81 | -87.21±21.64 | 0.954^b^ |
| SAT area, cm^2^ | 18.83±16.78 | 16.88±9.90 | 20.56±20.98 | 0.814^b^ |
| SAT area density, HU | -98.37±11.63 | -98.45±11.67 | -98.29±11.65 | 0.930^b^ |
| **Toxicity outcomes** |  |  |  |  |
| Any grade 3/4 toxicity | 84(46.9%) | 84(100%) | NA | NA |
| Grade 3/4 hematological toxicity | 70(39.1%) | 70(100%) | NA | NA |
| Grade 3/4 neutropenic fever toxicity | 55(30.7%) | 55(100%) | NA | NA |
| Grade 3/4 gastrointestinal toxicity | 13(7.3%) | 13(100%) | NA | NA |
| Grade 3/4 neuropathy | 9(5.0%) | 9(100%) | NA | NA |
| Other grade 3/4 toxicities | 19(10.6%) | 19(100%) | NA | NA |
| Hospitalization | 51(28.5%) | 51(100%) | NA | NA |
| Dose reductions/delays | 30(16.8%) | 30(100%) | NA | NA |

Abbreviations: DLBCL, diffuse large B-cell lymphoma; BMI, body mass index; BSA, body surface area; LDH lactate dehydrogenase; GCB, germinal center B cell; SM, skeletal muscle; VAT, visceral adipose tissue; SAT, subcutaneous adipose tissue.

^a^Student’s t test

^b^Mann–Whitney U test

^c^Chi-squared test

Supplementary Table S2. Baseline characteristics of patients with DLBCL by BMI.

| Variables | BMI < 25.0 | BMI ≥ 25.0 | P |
| --- | --- | --- | --- |
| No. patients | 116 | 63 | - |
| **Patient characteristics** | | | |
| Age, years | 56.88±14.09 | 57.10±12.43 | 0.887^b^ |
| BSA, m^2^ | 1.68±0.13 | 1.85±0.14 | <0.001^a^ |
| Ann Arbor stage |  |  | 0.600^c^ |
| I-II | 47(40.5%) | 23(36.5%) |  |
| III-IV | 69(59.5%) | 40(63.5%) |  |
| LDH>UPN | 65(56.0%) | 37(58.7%) | 0.728^c^ |
| GCB/non-GCB | 47/69 | 26/37 | 0.922^c^ |
| International Prognostic Index |  |  | 0.411^c^ |
| Low-risk (0-2 points) | 101(87.1%) | 52(82.5%) |  |
| High-risk (3-5 points) | 15(12.9%) | 11(17.5%) |  |
| Diabetes Mellitus | 11(9.5%) | 6(9.5%) | 0.993^c^ |
| Hyperlipidemia | 1(0.9%) | 3(4.8%) | 0.092^c^ |
| **Multi-slice body composition from L1 to L5(means)** | | | |
| SM volume, cm^3^ | 1657.42±454.14 | 1872.49±569.50 | 0.017^b^ |
| SM volume density, HU | 38.91±7.17 | 38.16±7.19 | 0.254^a^ |
| SM% | 36.22±13.54 | 26.50±8.75 | <0.001^b^ |
| **Toxicity outcomes** | | | |
| Any grade 3-4 | 56(48.3%) | 28(44.4%) | 0.624^c^ |
| Grade 3-4 hematological toxicities | 45(38.8%) | 25(39.7%) | 0.907^c^ |
| Grade 3-4 neutropenic fever | 36(31.0%) | 20(31.7%) | 0.922^c^ |
| Grade 3-4 GI toxicity | 8(6.9%) | 5(7.9%) | 0.798^c^ |
| Grade 3-4 neuropathy | 8(6.9%) | 1(1.6%) | 0.121^c^ |
| Other grade 3-4 toxicities | 14(12.1%) | 5(7.9%) | 0.391^c^ |
| Hospitalization | 34(29.3%) | 17(27.0%) | 0.742^c^ |
| Dose reductions/delays | 18(15.5%) | 12(19.0%) | 0.546^c^ |

Abbreviations: DLBCL, diffuse large B-cell lymphoma; BMI, body mass index; BSA, body surface area; LDH lactate dehydrogenase; GCB, germinal center B cell; SM, skeletal muscle.

Supplementary Table S3. Body composition characteristics of 74 patients with DLBCL at baseline and after four cycles of chemotherapy.

| Variables | **Baseline** | **After four cycles of R-CHOP** | **Longitudinal body composition changes (Δ)** |
| --- | --- | --- | --- |
|  |  |  |  |
| BMI, kg/m^2^ | 23.70±2.91 | 23.99±3.60 | 0.29±1.88 |
| SM volume, cm^3^ | 1709.19±510.31 | 1659.56±498.22 | -49.63±255.48 |
| SM volume density, HU | 38.23±7.28 | 38.54±9.07 | 0.31±6.50 |
| VAT volume, cm^3^ | 1640.64±700.96 | 1831.09±788.88 | 190.45±461.77 |
| VAT volume density, HU | -81.41±23.22 | -89.09±9.97 | -7.69±23.37 |
| SAT volume, cm^3^ | 1976.80±1024.53 | 2002.69±1065.55 | 25.89±608.36 |
| SAT volume density, HU | -95.43±11.56 | -94.32±27.67 | 1.11±28.31 |
| SM area, cm^2^ | 15.55±11.24 | 13.43±4.05 | -2.13±10.16 |
| SM area density, HU | 38.91±7.61 | 39.30±9.42 | 0.39±6.37 |
| VAT area, cm^2^ | 14.27±12.22 | 13.61±6.32 | -0.66±11.43 |
| VAT area density, HU | -86.80±10.53 | -90.34±10.12 | -3.54±9.11 |
| SAT area, cm^2^ | 16.77±15.12 | 15.73±8.12 | -1.05±13.08 |
| SAT area density, HU | -98.26±13.22 | -99.53±10.36 | -1.27±14.78 |

Abbreviations: DLBCL, diffuse large B-cell lymphoma; BMI, body mass index; SM, skeletal muscle; VAT, visceral adipose tissue; SAT, subcutaneous adipose tissue.

Supplementary Table S4. ORs (95% CIs) of toxicity for changes in body composition after four cycles of chemotherapy.

|  | Any grade 3/4 toxicity  (N=34) | Grade 3/4 hematologic toxicity (N=26) | Grade 3-4 neutropenia fever (N=20) | Grade 3/4 gastrointestinal toxicity (N=9) | Hospitalization (N=20) | Dose delay/reduction (N=10) |
| --- | --- | --- | --- | --- | --- | --- |
| ΔSM volume  (100 cm^3^ decrease) | 1.09(0.90,1.31) | 1.32(1.04,1.68)* | 1.36(1.05,1.76)* | 1.13(0.88,1.46) | 1.02(0.83,1.25) | 1.11(0.87,1.42) |
| ΔSM volume density (5 HU decrease) | 1.03(0.72,1.47) | 1.03(0.71,1.49) | 1.17(0.79,1.74) | 1.32(0.78,2.24) | 1.34(0.89,2.02) | 1.09(0.65,1.81) |
| ΔSM area  (1 unit decrease) | 1.00(0.96,1.05) | 1.01(0.97,1.06) | 1.01(0.96,1.08) | 1.01(0.93,1.09) | 1.02(0.96,1.08) | 1.04(0.99,1.09) |
| ΔSM area density  (1 unit decrease) | 1.03(0.95,1.11) | 1.02(0.95,1.10) | 1.04(0.96,1.13) | 1.06(0.95,1.18) | 1.07(0.98,1.16) | 1.06(0.95,1.17) |
| ΔVAT volume | 1.00(1.00,1.00) | 1.00(1.00,1.00) | 1.00(1.00,1.00) | 1.00(1.00,1.00) | 1.00(1.00,1.00) | 1.00(1.00,1.00) |
| ΔVAT volume density | 1.00(0.98,1.02) | 1.01(0.99,1.03) | 1.02(0.97,1.08) | 1.03(0.96,1.12) | 1.04(0.98,1.10) | 1.09(1.01,1.19) |
| ΔVAT area | 1.02(0.97,1.06) | 1.03(0.98,1.08) | 1.00(0.96,1.05) | 1.02(0.93,1.12) | 1.01(0.96,1.06) | 1.04(0.99,1.08) |
| ΔVAT area density | 1.04(0.98,1.09) | 1.04(0.98,1.10) | 1.00(0.94,1.06) | 1.01(0.93,1.09) | 1.01(0.95,1.07) | 1.07(0.98,1.16) |
| ΔSAT volume | 1.00(1.00,1.00) | 1.00(1.00,1.00) | 1.00(1.00,1.00) | 1.00(1.00,1.00) | 1.00(1.00,1.00) | 1.00(1.00,1.00) |
| ΔSAT area density | 1.05(1.00,1.11) | 1.03(0.99,1.08) | 1.02(0.98,1.06) | 1.00(0.98,1.02) | 1.04(0.99,1.09) | 1.01(0.99,1.02) |
| ΔSAT area | 1.00(0.97,1.04) | 1.01(0.97,1.04) | 1.01(0.96,1.06) | 1.01(0.96,1.05) | 1.00(0.96,1.05) | 1.02(0.98,1.06) |
| ΔSAT area density | 1.02(0.99,1.05) | 1.02(0.99,1.05) | 1.01(0.98,1.04) | 1.02(0.97,1.07) | 1.02(0.98,1.06) | 1.04(0.99,1.09) |

Abbreviations: OR, odds ratio; CI, confidence interval; SM, skeletal muscle; VAT, visceral adipose tissue; SAT, subcutaneous adipose tissue.

****P*<0.001

***P*<0.01

**P*<0.05


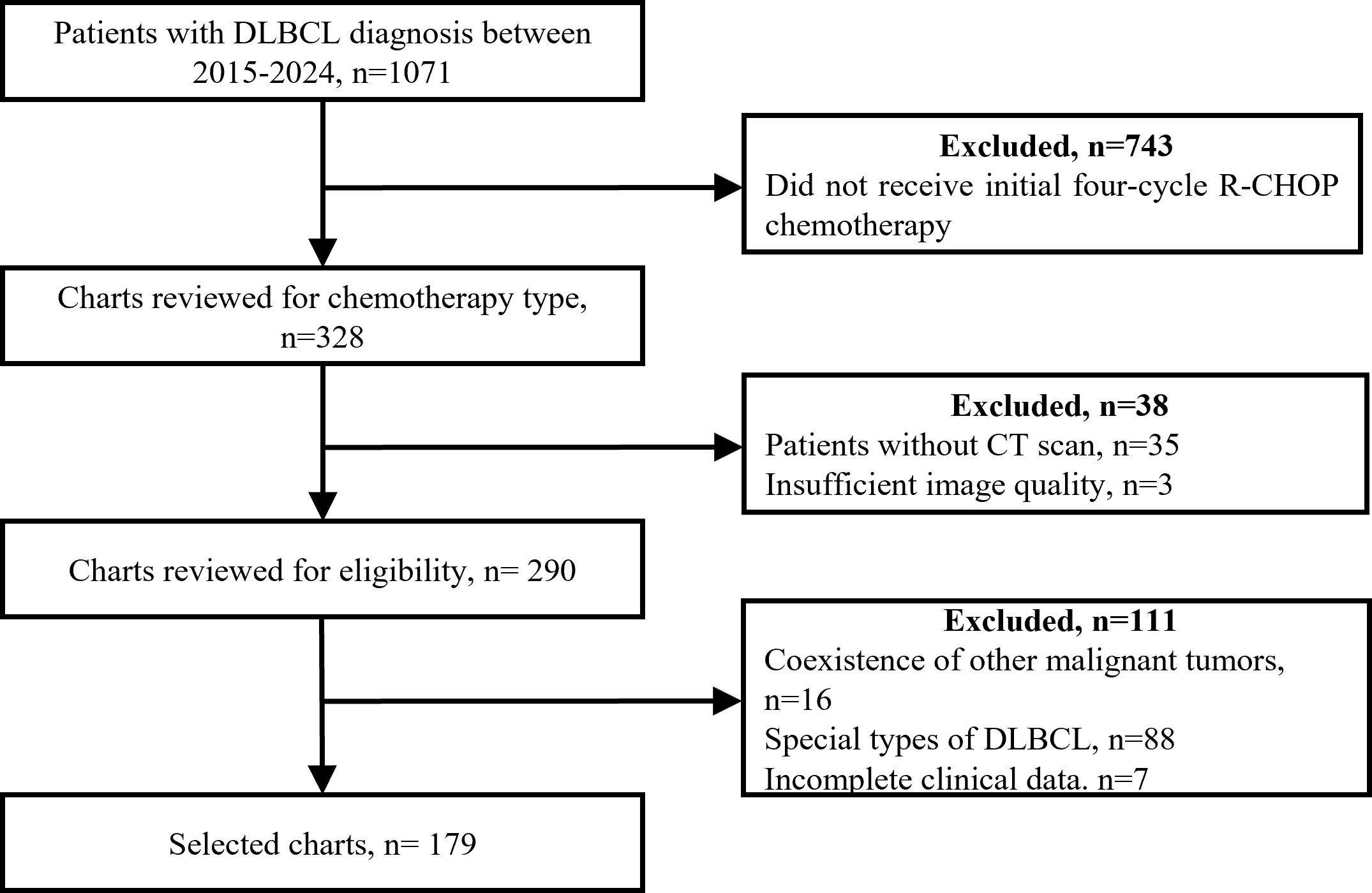


Supplementary Figure S1. Selection of participants for the current research.


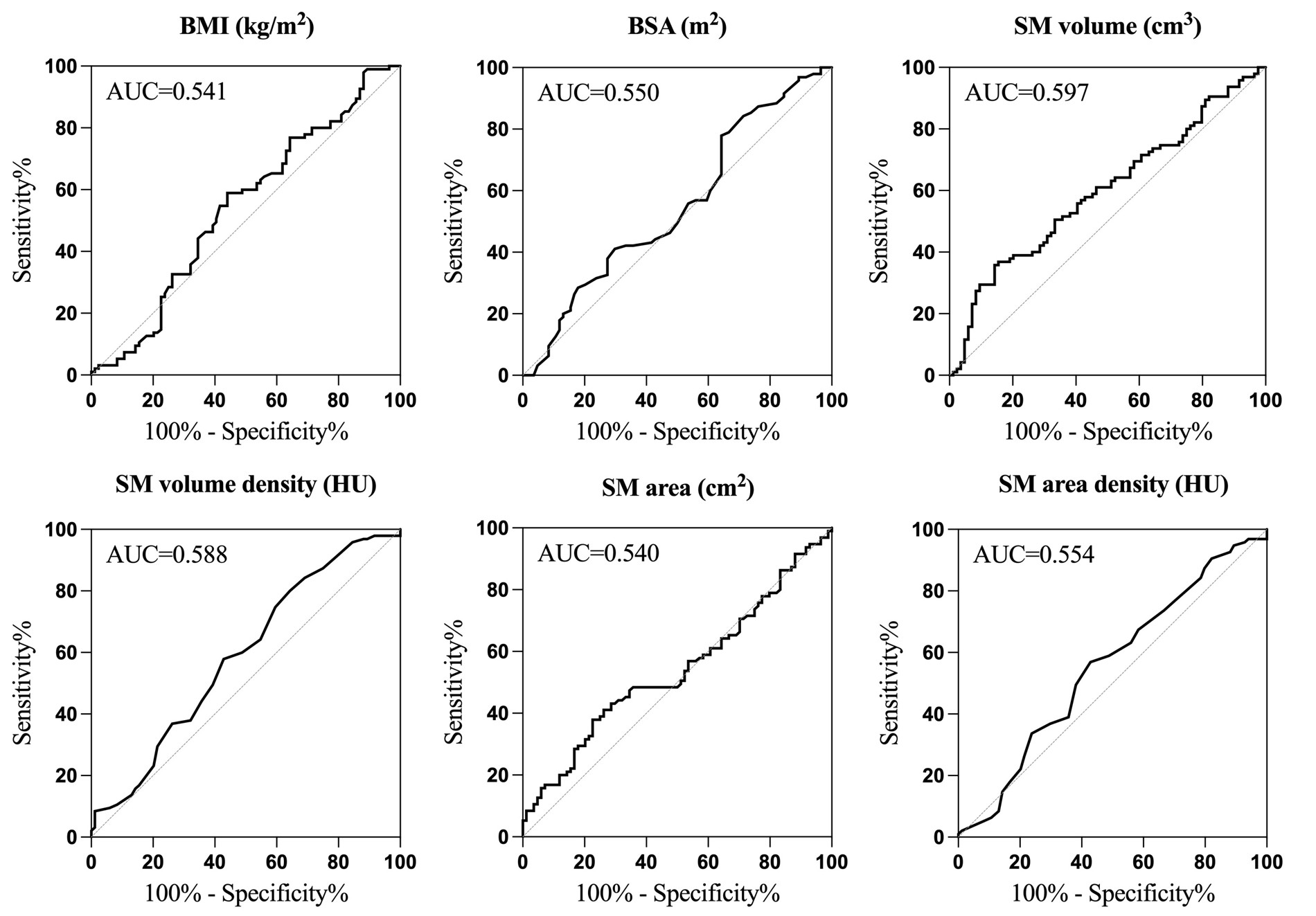


Supplementary Figure S2. ROCs for different body composition measures and any grade 3/4 toxicities.

Abbreviations: BMI, body mass index; BSA, body surface area; SM, skeletal muscle; HU, Hounsfield units.
